# Intelligent Flow Friction Estimation

**DOI:** 10.1155/2016/5242596

**Published:** 2016-04-03

**Authors:** Dejan Brkić, Žarko Ćojbašić

**Affiliations:** ^1^European Commission, DG Joint Research Centre (JRC), Institute for Energy and Transport (IET), Energy Security, Systems and Market Unit, Via Enrico Fermi 2749, 21027 Ispra, Italy; ^2^Faculty of Mechanical Engineering in Niš, University of Niš, Aleksandra Medvedeva 14, 18000 Niš, Serbia

## Abstract

Nowadays, the Colebrook equation is used as a mostly accepted relation for the calculation of fluid flow friction factor. However, the Colebrook equation is implicit with respect to the friction factor (*λ*). In the present study, a noniterative approach using Artificial Neural Network (ANN) was developed to calculate the friction factor. To configure the ANN model, the input parameters of the Reynolds Number (Re) and the relative roughness of pipe (*ε*/*D*) were transformed to logarithmic scales. The 90,000 sets of data were fed to the ANN model involving three layers: input, hidden, and output layers with, 2, 50, and 1 neurons, respectively. This configuration was capable of predicting the values of friction factor in the Colebrook equation for any given values of the Reynolds number (Re) and the relative roughness (*ε*/*D*) ranging between 5000 and 10^8^ and between 10^−7^ and 0.1, respectively. The proposed ANN demonstrates the relative error up to 0.07% which had the high accuracy compared with the vast majority of the precise explicit approximations of the Colebrook equation.

## 1. Introduction

To date, the Colebrook equation (1) is used as a mostly accepted standard for the calculation of fluid flow friction factor in pipes

(1) $$\begin{array}{c} \frac{1}{\sqrt{\lambda }}=-2\cdot \log_{10}\left(\;\frac{2.51}{Re\cdot \sqrt{\lambda }}+\frac{\varepsilon }{3.7\cdot D}\;\right), \end{array}$$

where *λ* is the Darcy friction factor (dimensionless); Re is Reynolds number (dimensionless), and *ε*/*D* is relative roughness of inner pipe surface (dimensionless).

The Colebrook equation is also somewhere known as the Colebrook-White equation or simply the CW equation [1]. Classifying the available data and those from experiment conducted in 1937 by himself and his professor White [2], Colebrook developed a curve fit which was describing transitional roughness, between the smooth and the rough turbulent zone [3]. The Colebrook equation is also considered as a proper base for the widely used Moody diagram with the exception of its laminar zone [4]. In other words, drawing his present famous diagram, Moody used Colebrook's equation for the whole turbulent zone and for the laminar zone defined by *λ* = 64/Re. The Moody chart or Moody diagram is a graph in nondimensional form that relates the Darcy friction factor (*λ*), the Reynolds number (Re), and the relative roughness (*ε*/*D*) for fully developed flow in a circular pipe. It can be used to determine pressure drop or flow rate in such pipes. Although the accuracy of empirical equation of Colebrook can be disputable, it is sometimes essential to produce a fast, accurate, and robust resolution of this equation, which is particularly necessary for the scientific intensive computations and very often for comparisons [5]. Unfortunately, the Colebrook equation suffers from being implicit with respect to the friction factor (*λ*). It cannot be rearranged to derive the friction factor directly with no approximate calculation. Many different strategies are used to calculate or to estimate the friction factor accurately [1, 6–8].

There are a group of studies investigating the use of Artificial Neural Network (ANN) to estimate the friction factor. For instance, the intelligent estimation of hydraulic resistance for Newtonian fluids has been investigated in some of recent studies [9–13]. For the other types of fluids used in agriculture, food engineering, petroleum engineering, and so forth, such as power-law, Bingham, Herschel-Bulkley, and other types of non-Newtonian fluids, the shown ANN cannot be used in the most cases. However, the developed methodology for training can be used with appropriate dataset or appropriate equations to produce relevant solution in such cases where the aforementioned ANN cannot be used [14–16]. Application of ANN for simulation of other types of friction factor rather than Colebrook, namely, Hazen–Williams friction coefficient for small-diameter polyethylene pipes, can also be found in the literature [17], while more recently other attempts of ANN usage for modeling friction factors in pipes have been reported [18, 19].

Nowadays, not only can the ANN approach be used in hydraulics and for simulation of fluid flow, but also it can be widely applied in the various branches of engineering, such as for the control systems [19, 20], as an auxiliary tool in medicine [21–25], a flow pattern indicator for gas-liquid flow in a microchannel [26], and an extension of structural mechanics tools for fast determination of structural response [27]. Also combined neurofuzzy systems (NFS) approach can be used for different purposes such as student modeling system, medical system, economic system, electrical and electronics system, traffic control, image processing and feature extraction, manufacturing and system modeling, forecasting and predictions, and social sciences [28].

## 2. Definition of the Problem

In the present study, in order to produce an efficient and accurate procedure for estimation of the flow friction factor (*λ*), an approach based on the computationally intelligent system was used. The Artificial Neural Network (ANN) for the solution of the problem is developed. The ANN models like the one shown here can be easily generated in the MATLAB software.

First, the raw datasets calculated using the Colebrook equation were used to train the ANN model and then the unknown friction factors (*λ*) were predicted by obtaining the ANN structure with a low relative error. In this paper, the empirical Colebrook equation (1) and its accurate iterative solution will be treated as “accurate by the default” or “absolutely accurate” (sign “=” is used, while for the approximations listed in Appendix sign “≈” is used).

Hydraulic resistance depends on the flow rate which is considered as the main problem in determination of the hydraulic flow friction factor (*λ*). For a pipe, the hydraulic resistance usually is expressed through the Darcy friction factor (*λ*) which is not a constant quantity. Friction factor (*λ*) is related to the flow rate or more precisely to the Reynolds number (Re) and the relative roughness (*ε*/*D*). In addition, both of them, the Reynolds number (Re) and the relative roughness (*ε*/*D*), are dependent on the flow rate. In fact, the Reynolds number (Re) is affected by flow velocity while the relative roughness (*ε*/*D*) depends on the thickness of a region of flow inside pipes, termed as boundary layer, which occurs closely to the inner surface of pipe wall [29, 30]. On the contrary, in this paper the relative roughness (*ε*/*D*) retains its classical definition, which implies it should not vary with the flow rate (it will be treated effectively as a geometric quantity and thus should be constant regardless of flow rate with the caveat that the flow is turbulent). Furthermore, it is obvious that changes of the hydraulic resistance in the turbulent zone are governed by the nonlinear law. In general, these hydraulic resistances in turbulent zone can be modeled as logarithmic-law or power-law [31]. The Colebrook equation belongs to the logarithmic-law.

As it was mentioned, the main problem of the Colebrook equation is related to its implicit form with respect to the friction factor (*λ*) which cannot be evaluated without the approximate calculation (the Colebrook equation is a transcendent function). Therefore, different strategies are used to find adequate solution for Colebrook equation: iterative solution (in the present study, it was assumed that values calculated by this method are highly accurate) [6, 7], use of plenty of available explicit approximations of the Colebrook equation derived by numerous mathematical or numerical approaches [6, 8, 32, 33], using some graphical interpretations such as the Moody diagram [4], and so forth.

It should be taken into account that the Moody diagram cannot be used as a reliable and accurate replacement for the Colebrook equation as its reading error can be even more than few percent [10, 34, 35]. Using iterative methods, namely, the Newton-Raphson, the friction factor (*λ*) can be calculated from the Colebrook equation with high accuracy where the convergence of 0.01% requires less than 7 iterations. This accuracy (0.01%) should not be confused with the accuracy of the explicit approximations of the Colebrook equation [36]. Reviewing the relevant literature, one can realize that the vast majority of these approximations are extremely accurate and they can be used instead of implicit Colebrook equation to calculate the friction factor (*λ*). However, the final maximal error caused by approximation should be estimated as the sum of the real maximal error of certain approximation and the error caused by iterative procedure.

The two most accurate explicit approximations with the relative errors up to 0.0026% and 0.0083% are those implied by Ćojbašić and Brkić [37]. Moreover, there are plenty of other approximations with the relative errors above 0.13% [6]. Indeed, use of the highly accurate approximations could complicate the fluid flow calculations. However, use of the advanced and powerful computers and codes can partially solve this problem and reduce the computational burden [38].

In this study, the implied ANN structure led to a low relative error compared to the accurate iterative solution. In addition, the computational burden used to run the applied ANN structure was equal or lower than that of explicit approximations, and it, especially, was less than that of the iterative solution of the original Colebrook equation, while the accuracy of the ANN approach remains significantly high.

## 3. Methodology

### 3.1. Preparation of the Dataset

In order to generate the training set for the ANN model, the Colebrook equation was solved iteratively. The iterative solution is used because the highly accurate solution of the friction factor (*λ*) was required, while in the meantime the computational burden was irrelevant since it was a onetime effort to prepare the training data. The training dataset can be efficiently prepared using the spreadsheet solvers, such as MS Excel which is used in the particular case presented here [6, 7]. In order to obtain the highest accuracy in the calculation using MS Excel, the iterative calculation should be enabled and the maximum number of iterations (it is set to 32,767 iterations which was the maximum number of cycles allowed by the software with the highest precision) has to be set [7].

In order to train the presented ANN model, input dataset (Electronic Appendix A: MS Excel spreadsheet with the set of 90 thousand combinations used for training of the Artificial Neural Network (ANN) (see Supplementary Material available online at http://dx.doi.org/10.1155/2016/5242596) involving 90,000 triplets was used in which the values of the Darcy friction factor (*λ*) were generated using values of the Reynolds number (Re) and the relative roughness (*ε*/*D*) ranged 5000–10^8^ and 10^−7^–0.1, respectively. In order to use input datasets, the values of the Reynolds number (Re) and the relative roughness (*ε*/*D*) had to be normalized. The used approach will be comprehensively explained in the next parts.

### 3.2. Structure and Training of the ANN

**Figure 1:**
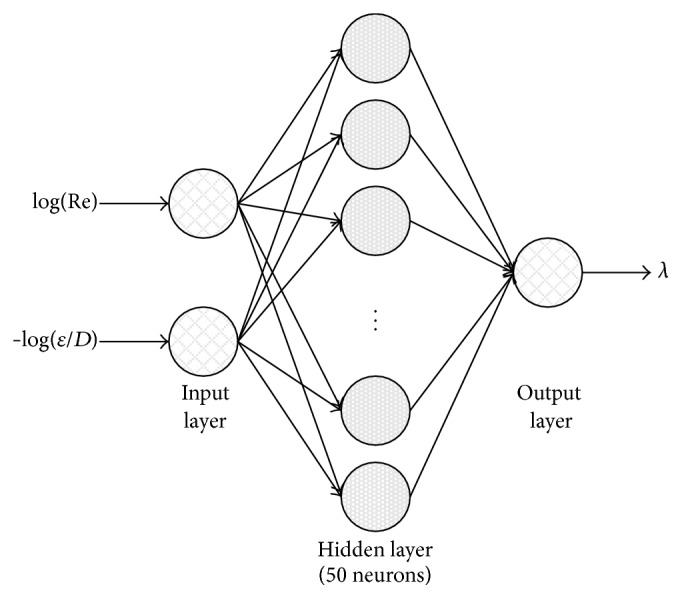
Structure of the proposed ANN.

The feedforward neural network structure which consists of three layers is used (Figure 1). The first, input layer has two neurons, the second, hidden layer has fifty neurons, and the third, output layer has one neuron, with a sigmoid transfer function in the hidden layer and a linear transfer function in the output layer.

In general, an ANN should be trained, or adapted, either before or during its use. The used ANN network was properly trained and validated by supervised offline training prior to network application in which the data obtained by the iterative solution of the Colebrook equation were applied.

Almost every neural network consists of a large number of simple processing elements that are variously called neurons, nodes, cells, or units, connected to other neurons by means of direct communication links, each with an associated weight and bias. The weights represent information being used by the net to produce output for given inputs. The most common feedforward net has two or more layers of processing units in the adjacent layers. Generally speaking, ANN is able to efficiently imitate functions and recognize patterns. They can be trained to solve a problem (ability to learn). The quality of this solution heavily depends on the quantity of available data for training and the structure of a network.

It should be underlined that the developed ANN (the generated ANN is attached as Electronic Appendix B to this paper; file ColebrookANN.mat) does not use the Colebrook equation for the calculation. It uses only the results produced by the Colebrook equation to establish its inner patterns. Every neural network is considered as a “Black box” system; therefore, it can be viewed in terms of its inputs and outputs without any knowledge about its internal working and inner components.

However, the main issue of the present network is related to the ranges of input parameter in which the relative roughness (*ε*/*D*) is extremely small as it ranged from 10^−7^ to 0.1, while another parameter, the Reynolds number (Re), is considerably large in the range of 2320 to 10^8^. This problem can prevent the ANN from being properly trained and it will lead to the less accurate results in application phase. Therefore, the raw input dataset should be normalized to provide the input data for the ANN with the approximately same order of magnitude.

In order to address this issue, the logarithmic transformation can be done where the Reynolds number (Re) and the relative roughness (*ε*/*D*) were replaced by log⁡(Re) and −log⁡(*ε*/*D*), respectively. These transformations translated (copied) input values into the new domain where log⁡(Re) is in range between 3.7 and 8 and −log⁡(*ε*/*D*) is in range between 1 and 6.5. Dataset set with the 90,000 combinations of the Reynolds number (Re), the relative roughness (*ε*/*D*), and related friction factor (*λ*) was prepared in MS Excel as already explained. Full prepared dataset was divided into training, validation, and testing subsets:

- The training sample (70%, 63,000 triplets) was presented to the ANN during the training,
- the validation sample (15%, 10,500 triplets) was used to measure generalization of the ANN, that is, to stop the training when the generalization does not improve anymore (i.e., this prevents the so-called “overfitting”),
- the testing sample (15%, 10,500 triplets) had no effect on the training and so it provided an independent measure of performance of the ANN during and after training.

**Figure 2:**
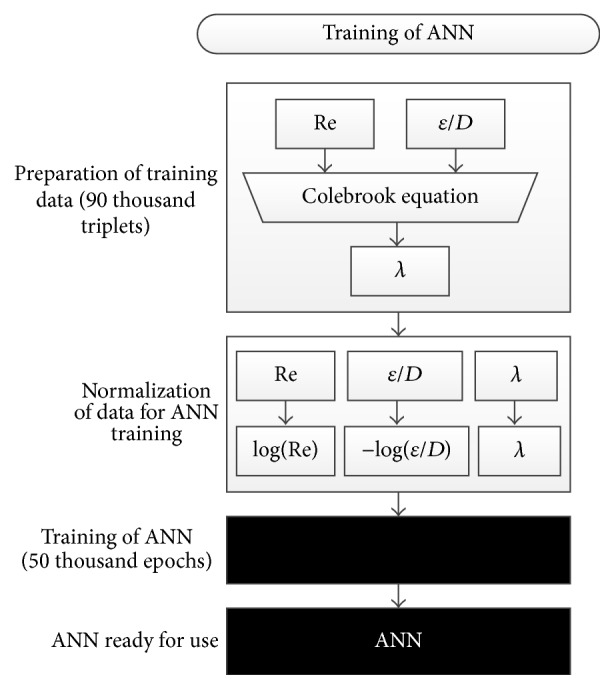
The scheme of training process of the ANN.

Inputs were normalized and used for the training of the ANN which is indicated in Figure 1. The concept of the training process is shown in Figure 2. The Neural Network Toolbox of MATLAB software was used to simulate the proposed ANN for the shown flow friction problem.

### 3.3. Use of the ANN

**Figure 3:**
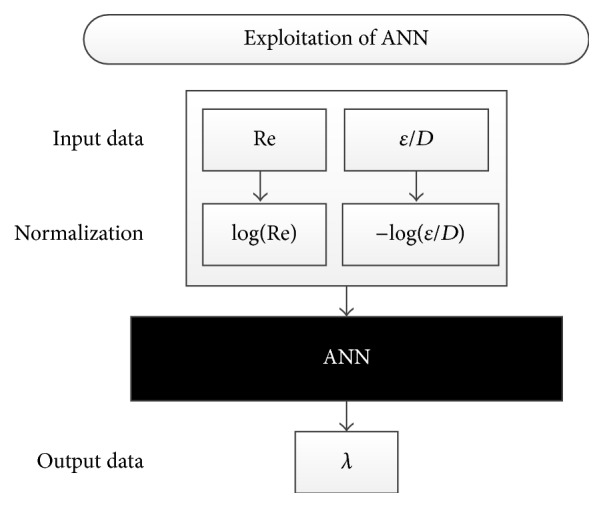
Exploitation of the ANN.

When the training process with 90,000 inputs/output combinations of data was finalized, the generated ANN was saved under the name of “ColebrookANN” for later uses. In such a way, the ANN can be further used for the accurate estimation of the flow friction factor (*λ*). The Colebrook equation was used for the training process of the ANN model. Then, the generated ANN will use inputs and produce results that follow this pattern from the learning phase for any unknown combination of inputs. The phase of exploitation of network is shown in Figure 3.

For the presented ANN, the process of training lasted few hours. Afterwards, the ANN can be used to estimate flow friction factor (*λ*), accurately. This can be carried out using MATLAB software by loading network previously saved with the name “ColebrookANN” using command:

 
*load ColebrookANN.mat*

The hydraulic friction factor (*λ*) can be evaluated using single line in MATLAB:

 
*lambda=sim(ColebrookANN, [log10(Re); −log10(RPR)]),*

where Re denotes the values for the Reynolds number (Re) while RPR denotes relative roughness (*ε*/*D*), that is, Relative Pipe Roughness (RPR), in order to avoid Greek letters in the code. Due to MATLAB exquisite matrix handling capabilities, the sets of pairs of input data can be prepared in one row by multiple columns vector variables of the Re and the RPR. In this case the MATLAB produces vector lambda involving the calculated friction factors (*λ*) for each input data pair in fraction of time, even for the large datasets.

In order to determine the hydraulic friction factor (*λ*) using ANN, the sufficiently large training dataset was used which was in contrast to other published results in this field [9–13]. The proposed network can outperform even the most accurate approximations to the Colebrook equation.

## 4. Results and Discussion

### 4.1. Model Performance

In order to examine the performance of a model, approximation quality, model complexity, and model interpretability should be addressed. In fact, the approximation/prediction error is often used as an assessment criterion. There are different criteria in the literature to assess the model performance. It is possible that the worst case or the average deviation is crucial [39, 40].

**Figure 4:**
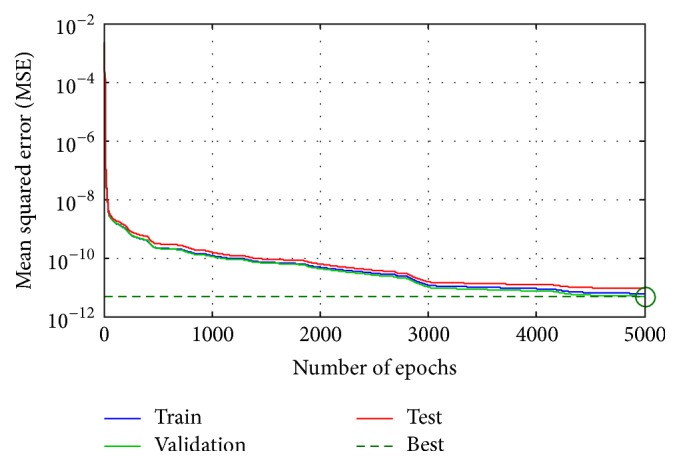
The Mean Squared Error (MSE) during the process of training of the proposed ANN.

For training of the presented ANN, the back propagation Levenberg-Marquardt algorithm was used, while the Mean Squared Error (MSE) was used as performance measure during the training phase. The values of MSE for this ANN structure were calculated to be 10^−12^ after 5,000 epochs of training (Figure 4). The main goal was to minimize the performance function, in this case MSE function, which is defined as

(2) $$\begin{array}{c} \text{MSE}=\frac{1}{n}\overset{n}{\underset{k=1}{\sum }}e_{k}^{2}=\frac{1}{n}\overset{1}{\underset{k=1}{\sum }}{\left(\;t_{k}-y_{k}\;\right)}^{2}, \end{array}$$

where *n* denotes number of samples, *e*_*k*_ denotes neural network error, and *t*_*k*_ denotes target values, while *y*_*k*_ are network output values. The training algorithm used in all cases was Levenberg-Marquardt algorithm [41], where network weights *w* are updated by the equation **w**_*k*+1_ = **w**_*k*_ − (**J**_*k*_^*T*^ **J**_*k*_ + *μ* **I**)^−1^ **J**_*k*_ *e*_*k*_ and which is based on the approximation of Hessian matrix **H** = **J** **J** ^*T*^ + *μ* **I**, where **J** denotes Jacobian matrix, **I** denotes identity matrix, and *μ* is always positive so-called combination coefficient. The Levenberg-Marquardt algorithm was selected as being stable, fast, and reliable.

The training of the proposed ANN structure was done through 5,000 epochs. The Mean Squared Error (MSE) of this ANN structure was calculated to be 10^−12^ after which there was no further tendency to decrease. In addition, the same results were obtained with the tested ANN structures involving 100 neurons in a hidden layer and with the two hidden layers containing 50 neurons in each of them. However, the tested ANN structure with 30 neurons in one hidden layer resulted in a lower accuracy in comparison with the former tested structures, even after 10,000 epochs of training.

### 4.2. Accuracy of the Estimated Results

For the purpose of comparison, it is better to use the relative error than the Mean Squared Error (MSE) which was used during the training process of the proposed ANN. The maximum relative error of the proposed feedforward ANN structure, with one hidden layer containing 50 neurons, compared with the iterative solution of the Colebrook equation, was up to 0.07% (Table 1).

It should be taken into account that there are three levels of the accuracy [36, 41]:

1. The first level is related to the nature of the Colebrook equation which is an empirical relation (in fact, there is a possibility of using other equations with higher accuracy, and accordingly the showed methodology can be used in order to develop the appropriate ANN for such a case).
2. The second level explains the accuracy related to the solution of the Colebrook equation; the Colebrook equation can be solved precisely using the iterative procedure (in this paper, the term “accurate by default” or “absolutely accurate” and the related error can be neglected in many cases).
3. The third one is related to the proposed ANN structures and relevant approximations which can be used to avoid iterative procedure; their errors can be estimated and compared with the error of iterative solution (obtained error of the suggested ANN structure belongs to the third category).

**Figure 5:**
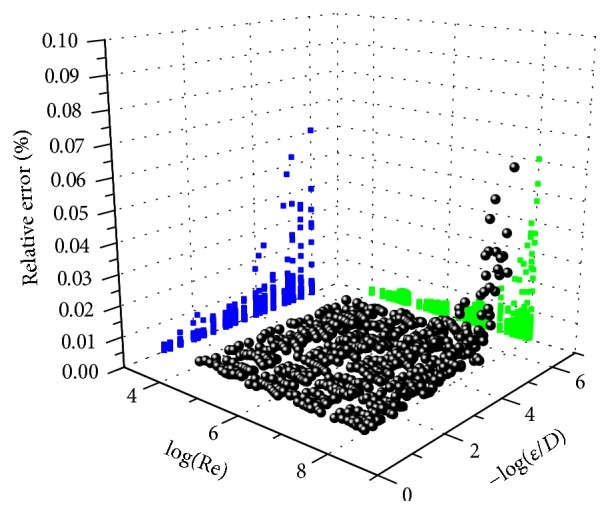
Distribution of the estimated error produced by the ANN compared with the Colebrook equation in normalized domain which is suitable for training of the ANN (verification in MATLAB).

**Figure 6:**
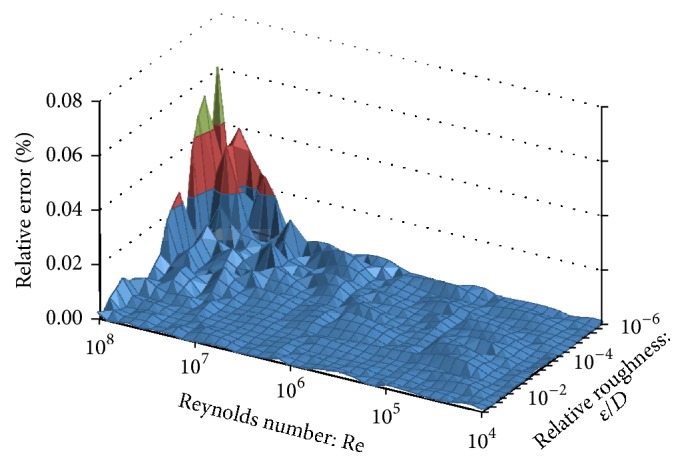
Distribution of the estimated error produced by the ANN compared with the Colebrook equation (verification in MS Excel).

The relative error of friction factor estimated through the proposed ANN structure in this is up to 0.07% (Figures 5 and 6). This means that proposed ANN approach can be used not only as extremely accurate approach, but also as a computationally effective one.

Furthermore, to some extent, an increase in the complexity of the ANN structure would augment its potential to produce even more accurate results. Hence, the right balance of accuracy and complexity is necessary during the network design phase. Additionally, accuracy depends on the quantity of terms in the training set. The complexity of network in the phase of exploitation is relatively unimportant since the ANN is a sort of “black box.” It can produce outputs for inputs and its inner complexity is not crucial [47, 48].

Users would easily apply the ANN without any difficulty due to its structure complexity, in contrast to use of the approximate formulas [38]. The same circumstances of comfort can be experienced by users applying the prepared computer codes for the approximate formulas. Users will be able to enter input data into a program and a computer should be able further to produce outputs without any inconvenience.

According to Figures 5 and 6, the relative error is not equally distributed over the entire practical range of the Reynolds number (Re) and the relative roughness (*ε*/*D*). The same situation with this distribution of the error would occur for the explicit approximations as shown by Brkić [6, 7] and Winning and Coole [33]. The relative error produced by the ANN is accumulated in the zone with small values of the relative roughness (*ε*/*D*) and the high values of the Reynolds number (Re). The distribution of the relative error is also shown in Table 1. According to Table 1 the maximum relative error was calculated to be 0.0606% for Re = 10^8^ and *ε*/*D* = 10^−6^.

### 4.3. Comparative Analysis

**Figure 7:**
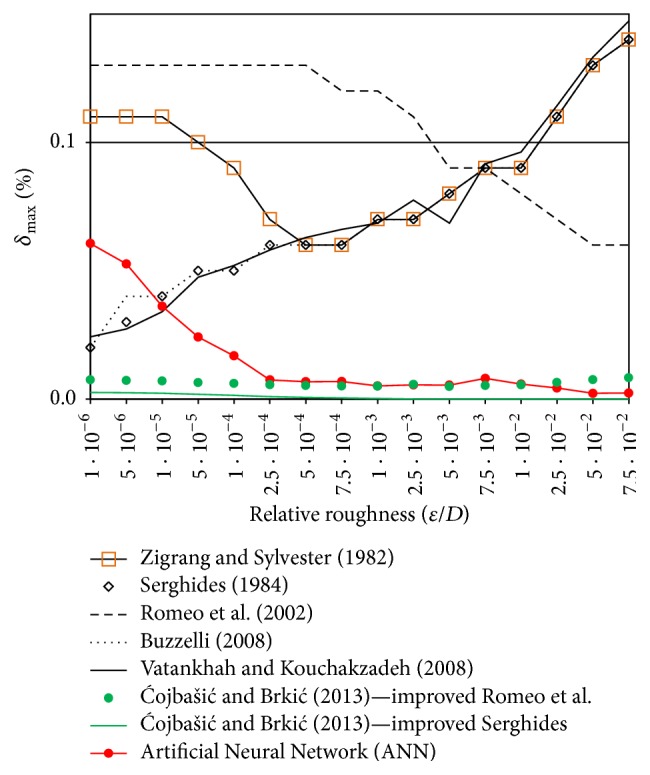
Maximal relative error produced by ANN compared with the seven most accurate explicit approximations of Colebrook equation where *ε*/*D* is used for *x*-axis.

Having looked at the existing approximations of Colebrook equation [6, 7], one can obviously realize that the available explicit approximations of the Colebrook equation are either inaccurately simple or intricately accurate. In fact, the complexity of explicit approximations (e.g., approach with the Lambert W-function [8, 49]) was considered as a serious issue few decades ago when pocket calculators were widely used [38]. Nowadays, even the very complex approximations can be easily used in computer codes. In the study conducted by Brkić [6], it was concluded that the five most available approximations from the literature had the maximum relative error up to 0.15%. These approximations were suggested by Zigrang and Sylvester [46], Serghides [42], Romeo et al. [43], Buzzelli [45], and Vatankhah and Kouchakzadeh [44] (even more accurate approximations are shown in Vatankhah [50] where their accuracy is comparable with accuracy of approximations shown in Ćojbašić and Brkić [37]). Furthermore, Ćojbašić and Brkić [37] applied genetic algorithm optimization technique (also genetic technique are used in [51, 52]). This technique improved two of these accurate approximations suggested by Serghides [42] and Romeo et al. [43] to reach even extreme level of accuracy with the relative error up to 0.0026% and 0.0083%, respectively. All mentioned explicit approximations are listed in Appendix of this paper (they are also attached to this paper as Electronic Appendix C (PDF file with all approximations of the Colebrook equation mentioned through text with their MATLAB codes and MS Excel codes)). The accuracy of the proposed ANN in the present work was compared with accuracy of these approximations which is shown in Figure 7 where relative roughness (*ε*/*D*) is used as the base for the *x*-axis of the diagram. Moreover, in Table 2, the Reynolds number (Re) is used as the base. This means that, in the case of using relative error of the presented ANN from Figure 7, the maximum value of the relative error can be chosen from each column of Table 1, while, in the case of using of Table 2, the maximum value of the relative error can be chosen from each row of Table 1.

The results of comparative analysis which were reported in Figure 7 revealed that the implied ANN structure could outperform the vast majority of the most accurate approximations in the large area of data domain. In addition, the suggested ANN structure in this study might be used with the most accurate explicit approximations of the Colebrook equation implied by Ćojbašić and Brkić [37], Romeo et al. [43], Buzzelli [45], Serghides [42], Zigrang and Sylvester [46], and Vatankhah and Kouchakzadeh [44]. The maximum relative errors for these approximations were evaluated to be 0.0026%, 0.13%, 0.14%, 0.14%, 0.14%, and 0.15%, respectively.

## 5. Conclusion

In order to evaluate the friction factor, the sophisticated ANN model was developed. The model includes three layers of input, hidden, and output neurons with 2, 50, and 1 neurons, respectively. The trained ANN is able to predict friction factor (*λ*) with the relative error of less than 0.07%. Based on the performed comparative analysis, the developed ANN produces the lowest relative error in comparison with most of accurate explicit approximations of the Colebrook equation. Furthermore, to deal with the low accuracy of the Colebrook equation or to facilitate for specific needs, the suggested ANN structure could be trained using some of the other available precise approximations or experimental data [53, 54] (although each new training will produce different inner pattern among neurons [55], the final estimation of friction factor will remain with almost the same level of accuracy) and even using combination of these for different parts of input domains which could be considered as significant advantage [56]. For these reasons, this suggested ANN structure in the present study would be worthwhile to solve flow problems involving repetitive calculations of the friction factor (*λ*). An important disadvantage might be the fact that significant number of training patterns is required to obtain accuracy level presented in this paper, but this would be with limited impact since the problem can be overwhelmed with onetime effort.

In our approach we tried to keep the solution simple and provide single neural network that covers the whole range of inputs, but further interesting research direction would be to design several networks covering parts of input spaces and working in conjunction possibly providing improved accuracy and sacrificing simplicity of the solution. Also, following our own results and results of others regarding application of other techniques of computational intelligence for the same problem, the ANN presented here could potentially be cross-fertilized with them in an attempt to improve results, where primarily genetic optimization of the network structure might be promising.

## Supplementary Material

Electronic Appendix A contains MS Excel file, containing set of 90 thousand combinations used for training, validation and testing of the Artificial Neural Network (ANN). Electronic Appendix B is the Artificial Neural Network (ANN) generated in MathWorks MATLAB. Electronic Appendix C is PDF file with all approximations of the Colebrook equation mentioned through text with their MathWorks MATLAB codes and MS Excel codes.

## Figures and Tables

**Table 1:** Relative error of friction factor produced by the shown ANN over the practical domain of the relative roughness (*ε*/*D*) and the Reynolds number (Re).

Relative error (%)	Relative roughness (*ε*/*D*)									
Reynolds number (Re)	10^−6^	5 · 10^−6^	10^−5^	5 · 10^−5^	10^−4^	5 · 10^−4^	10^−3^	5 · 10^−3^	10^−2^	5 · 10^−2^
10^4^	0.00134	0.00088	0.00031	0.00017	0.00123	0.00141	0.00041	0.00099	0.00096	0.00069
5 · 10^4^	0.00102	0.00174	0.00080	0.00096	0.00220	0.00163	0.00247	0.00063	0.00224	0.00124
10^5^	0.00114	0.00145	0.00125	0.00356	0.00099	0.00384	0.00097	0.00117	0.00104	0.00076
5 · 10^5^	0.00181	0.00032	0.00287	0.00084	0.00047	0.00090	0.00028	0.00011	0.00055	0.00064
10^6^	0.00163	0.00246	0.00126	0.00073	0.00419	0.00440	0.00176	0.00190	0.00023	0.00053
5 · 10^6^	0.00449	0.00672	0.00207	0.00377	0.00012	0.00077	0.00071	0.00031	0.00038	0.00074
10^7^	0.00126	0.00054	0.00417	0.00527	0.00005	0.00089	0.00015	0.00033	0.00063	0.00186
5 · 10^7^	0.01946	0.00382	0.00490	0.00835	0.00260	0.00174	0.00011	0.00071	0.00038	0.00022
10^8^	0.06060	0.05266	0.03614	0.02413	0.01682	0.00410	0.00165	0.00544	0.00579	0.00068

**Table 2:** Maximal relative error produced by the ANN compared with the seven most accurate explicit approximations of Colebrook equation; the Reynolds number (Re) is used as the base.

Maximal relative error (%)								
Reynolds number (Re)	(a)	(b)	(c)	(d)	(e)	(f)	(g)	(h)
10^4^	0.00141	0.00074	0.00569	0.12272	0.13563	0.13453	0.13301	0.13313
5 · 10^4^	0.00247	0.00219	0.00574	0.14112	0.13784	0.11047	0.13736	0.13736
10^5^	0.00384	0.00246	0.00698	0.14467	0.13812	0.10281	0.13793	0.13793
5 · 10^5^	0.00287	0.00250	0.00802	0.14712	0.13841	0.08915	0.13839	0.13839
10^6^	0.00440	0.00235	0.00816	0.14727	0.13846	0.08426	0.13845	0.13845
5 · 10^6^	0.00672	0.00167	0.00826	0.14725	0.13850	0.07315	0.13850	0.13850
10^7^	0.00527	0.00122	0.00828	0.14722	0.13851	0.06754	0.13850	0.13850
5 · 10^7^	0.01946	0.00022	0.00829	0.14718	0.13851	0.04876	0.13851	0.13851
10^8^	0.06060	0.00005	0.00829	0.14718	0.13851	0.04841	0.13851	0.13851

(a)-Artificial Neural Network (ANN).

(b)-Ćojbašić and Brkić [37]-Improved Serghides [42]; (A.7).

(c)-Ćojbašić and Brkić [37]-Improved Romeo et al. [43]; (A.6).

(d)-Vatankhah and Kouchakzadeh [44]; (A.2).

(e)-Buzzelli [45]; (A.1).

(f)-Romeo et al. [43]; (A.3).

(g)-Serghides [42]; (A.4).

(h)-Zigrang and Sylvester [46]; (A.5).

## Appendix

Approximations of the Colebrook equation for flow friction used in this paper are as follows (MATLAB and MS Excel codes for the shown approximations are listed in Electronic Appendix C of this paper) [*λ*,  Re,  *ε*/*D* are with the same meaning as in (1) of this paper while *A*_1–16_ are auxiliary terms]:

(i) Buzzelli approximation [45]:
  (A.1) 1λ≈A1−A1+2·log10⁡A2/Re1+2.18/A2,A1≈0.774·ln⁡Re−1.411+1.32·ε/D,A2≈13.7·εD·Re+2.51·A1.
(ii) Vatankhah and Kouchakzadeh [44, 50, 57]:
  (A.2) 1λ≈0.8686·ln0.4587·ReA3−0.31A4,A3≈0.124·Re·εD+ln0.4587·Re,A4≈A3A3+0.9633.
(iii) Romeo et al. approximation [43]:
  (A.3) 1λ≈−2·log10⁡13.7065·εD−5.0272Re·A5,A5≈log10⁡13.827·εD−4.567Re·A6,A6≈log10⁡17.7918·εD0.9924+5.3326208.815+Re0.9345.
(iv) Serghides approximation [42]:
  (A.4) 1λ≈A7−A8−A72A9−2·A8+A7,A7≈−2·log10⁡13.7·εD+12Re,A8≈−2·log10⁡13.7·εD+2.51·A7Re,A9≈−2·log10⁡13.7·εD+2.51·A8Re.
(v) Zigrang and Sylvester approximation [46, 58]:
  (A.5) 1λ≈−2·log10⁡13.7·εD−5.02Re·A10,A10≈log10⁡13.7·εD−5.02Re·A11,A11≈log10⁡13.7·εD+13Re.
(vi) Ćojbašić and Brkić approximation [37, 43]:
  (A.6) 1λ≈−2·log10⁡13.7106·εD−5Re·A12,A12≈log10⁡13.8597·εD−4.795Re·A13,A13≈log10⁡17.646·εD0.9685+4.9755206.2795+Re0.8759.
(vii) Ćojbašić and Brkić approximation [37, 42]:
  (A.7) 1λ≈A14−A15−A142A16−2·A15+A14,A14≈−2·log10⁡13.71·εD+12.585Re,A15≈−2·log10⁡13.71·εD+2.51·A14Re,A16≈−2·log10⁡13.71·εD+2.51·A15Re.

## Additional Points

Software packages used for this research are MS Excel ver. 2007 and MATLAB R2010a by MathWorks. The paper is registered in the internal system for publication PUBSY of the Joint Research Centre of the European Commission under no. JRC100455.
