# Supplementary material for: Intelligent Flow Friction Estimation
Source: Comput Intell Neurosci. 2016 Apr 3;2016:5242596. doi: 10.1155/2016/5242596 (PMC4834174; doi:10.1155/2016/5242596)
Supplement: Supplementary file 1 — Electronic Appendix A contains MS Excel file, containing set of 90 thousand combinations used for training, validation and testing of the Artificial Neural Network (ANN). Electronic Appendix B is the Artificial Neural Network (ANN) generated in MathWorks MATLAB. Electronic Appendix C is PDF file with all approximations of the Colebrook equation mentioned through text with their MathWorks MATLAB codes and MS Excel codes. [file 5242596.f1.zip › El._Appendix_C_CIN_1555776.pdf]

Approximations of the Colebrook equation for flow friction mentioned in text
MATLAB and MS Excel codes for the shown approximations
Re-Reynolds number (dimensionless),
ε/D-Relative roughness (dimensionless),
λ-Darcy friction factor (dimensionless)
(Re-Reynolds number, RelEpsilon-Relative roughness; in MS Excel: ‘A1: Re and B1: RelEpsilon’)

1. Buzzelli approximation

$$\left.\begin{aligned}
\frac{1}{\sqrt{\lambda}} &\approx A_1 - \left(\frac{A_1 + 2\cdot\log_{10}\left(\frac{A_2}{\mathrm{Re}}\right)}{1+\frac{2.18}{A_2}}\right)\\
A_1 &\approx \frac{\left(0.774\cdot\ln(\mathrm{Re})\right)-1.41}{\left(1+1.32\cdot\sqrt{\frac{\varepsilon}{D}}\right)}\\
A_2 &\approx \frac{1}{3.7}\cdot\frac{\varepsilon}{D}\cdot\mathrm{Re}+2.51\cdot A_1
\end{aligned}\right\}$$

MATLAB:

```
function Lambda=Lambda(Re,RelEpsilon)
A1 = ((0.774.*log(Re))-1.41)./(1+1.32.*sqrt(RelEpsilon));
A2 = (RelEpsilon./3.7).*Re+2.51.*A1;
Lambda = (A1-((A1+2.*log10(A2./Re))/(1+2.18./A2))).^-2;
```

MS Excel:

```
=(((0.774*LN(A1))-1.41)/(1+1.32*SQRT(B1)))-((((((0.774*LN(A1))-
1.41)/(1+1.32*SQRT(B1))))+2*LOG10(((1/3.7)*B1*A1+2.51*(((0.774*LN(A1))-
1.41)/(1+1.32*SQRT(B1))))/A1))/(1+2.18/(B1*(1/3.7)*A1+2.51*((((0.774*LN(A1))-
1.41)/(1+1.32*SQRT(B1))))))
```
cell C1

```
=POWER(1/C1,2)
```

Buzzelli, D. (2008). “Calculating friction in one step.” *Mach. Des.*, 80(12), 54–55.

2. Vatankhah and Kouchakzadeh

$$
\left.\begin{aligned}
&\frac{1}{\sqrt{\lambda}} \approx 0.8686 \cdot \ln\left(\frac{0.4587 \cdot \mathrm{Re}}{\left(A_3 - 0.31\right)^{A_4}}\right)\\
&A_3 \approx 0.124 \cdot \mathrm{Re} \cdot \frac{\varepsilon}{D} + \ln\left(0.4587 \cdot \mathrm{Re}\right)\\
&A_4 \approx \frac{A_3}{A_3 + 0.9633}
\end{aligned}\right\}
$$

MATLAB:

```
function Lambda=Lambda(Re,RelEpsilon)
G = 0.124.*Re.*RelEpsilon+log(0.4587.*Re);
Lambda = (0.8686.*log(0.4587.*Re./(G-0.31).^(G/(G+0.9633)))).^-2;
```

MS Excel:

=0.8686*LN(0.4587*A1/POWER((0.124*A1*B1+LN(0.4587*A1))-0.31,(0.124*A1*B1+LN(0.4587*A1))/((0.124*A1*B1+LN(0.4587*A1))+0.9633)))

cell C1

=POWER(1/C1,2)

Vatankhah, A.R., Kouchakzadeh, S. (2008). “Discussion of ‘Turbulent flow friction factor calculation using a mathematically exact alternative to the Colebrook-White equation.’ by Jagadeesh R. Sonnad and Chetan T. Goudar.” *J. Hydraul. Eng. ASCE*, 134(8), 1187. http://dx.doi.org/10.1061/(ASCE)0733-9429(2008)134:8(1187)

3. Romeo, Royo and Monzón approximation

$$\left.\begin{array}{l}\frac{1}{\sqrt{\lambda}} \approx -2\cdot\log_{10}\left(\frac{1}{3.7065}\cdot\frac{\varepsilon}{D}-\frac{5.0272}{\mathrm{Re}}\cdot A_5\right)\\ A_5 \approx \log_{10}\left(\frac{1}{3.827}\cdot\frac{\varepsilon}{D}-\frac{4.567}{\mathrm{Re}}\cdot A_6\right)\\ A_6 \approx \log_{10}\left(\left(\frac{1}{7.7918}\cdot\frac{\varepsilon}{D}\right)^{0.9924}+\left(\frac{5.3326}{208.815+\mathrm{Re}}\right)^{0.9345}\right)\end{array}\right\}$$

MATLAB:

```
function Lambda=Lambda(Re,RelEpsilon)
Lambda = (-2.*log10((RelEpsilon./3.7065)-
(5.0272./Re).*log10((RelEpsilon./3.827)-
(4.567./Re).*log10((RelEpsilon./7.7918).^0.9924+(5.3326./(208.815+Re))
.^0.9345)))).^-2;
```

MS Excel:

=-2*LOG10(B1*(1/3.7065)-5.0272/A1*LOG10(B1*(1/3.827)-
(4.567/A1)*LOG10(POWER(B1*(1/7.7918),0.9924)+POWER(5.3326/(208.815+A1),0.9345)))) cell C1

=POWER(1/C1,2)

Romeo, E., Royo, C., Monzón, A. (2002). “Improved explicit equations for estimation of the friction factor in rough and smooth pipes.” *Chem. Eng. J.*, 86(3), 369–374. http://dx.doi.org/10.1016/S1385-8947(01)00254-6

4. Serghides approximation

$$\left.\begin{array}{l}\frac{1}{\sqrt{\lambda}} \approx A_7 - \frac{\left(A_8 - A_7\right)^2}{A_9 - 2 \cdot A_8 + A_7} \\ A_7 \approx -2 \cdot \log_{10}\left(\frac{1}{3.7} \cdot \frac{\varepsilon}{D} + \frac{12}{\mathrm{Re}}\right) \\ A_8 \approx -2 \cdot \log_{10}\left(\frac{1}{3.7} \cdot \frac{\varepsilon}{D} + \frac{2.51 \cdot A_7}{\mathrm{Re}}\right) \\ A_9 \approx -2 \cdot \log_{10}\left(\frac{1}{3.7} \cdot \frac{\varepsilon}{D} + \frac{2.51 \cdot A_8}{\mathrm{Re}}\right)\end{array}\right\}$$

MATLAB:

```
function Lambda=Lambda(Re,RelEpsilon)
A7 = -2*log10(RelEpsilon./3.7+12./Re);
A8 = -2*log10(RelEpsilon./3.7+2.51.*A7./Re);
A9 = -2*log10(RelEpsilon./3.7+2.51.*A8./Re);
Lambda = (A7-((A8-A7).^2)/(A9-2.*A8+A7)).^-2;
```

MS Excel:

```
=POWER(-2*LOG10(B1*(1/3.7)+12/A1)-POWER(-2*LOG10(B1*(1/3.7)+2.51*(-
2*LOG10(B1*(1/3.7)+12/A1))/A1)--2*LOG10(B1*(1/3.7)+12/A1),2)/((-
2*LOG(B1*(1/3.7)+(2.51*(-2*LOG10(B1*(1/3.7)+(2.51*(-
2*LOG10(B1*(1/3.7)+12/A1))/A1)))/A1)))-2*(-2*LOG10(B1*(1/3.7)+2.51*(-
2*LOG10(B1*(1/3.7)+12/A1))/A1))+(-2*LOG10(B1*(1/3.7)+12/A1))),-2)
```

Serghides, T.K. (1984). "Estimate friction factor accurately." *Chem. Eng. (New York)*, 91(5), 63–64.

5. Zigrang and Sylvester approximation

$$\left.\begin{array}{l}\frac{1}{\sqrt{\lambda}} \approx -2 \cdot \log_{10}\left(\frac{1}{3.7} \cdot \frac{\varepsilon}{D} - \frac{5.02}{\mathrm{Re}} \cdot A_{10}\right) \\ A_{10} \approx \log_{10}\left(\frac{1}{3.7} \cdot \frac{\varepsilon}{D} - \frac{5.02}{\mathrm{Re}} \cdot A_{11}\right) \\ A_{11} \approx \log_{10}\left(\frac{1}{3.7} \cdot \frac{\varepsilon}{D} + \frac{13}{\mathrm{Re}}\right)\end{array}\right\}$$

MATLAB:

```
function Lambda=Lambda(Re,RelEpsilon)
Lambda = (-2.*log10((RelEpsilon./3.7)-
(5.02./Re)*log10((RelEpsilon./3.7)-
(5.02./Re)*log10(RelEpsilon./3.7+13./Re)))).^-2;
```

MS Excel:

=-2*LOG10((1/3.7)*B1-(5.02/A1)*LOG10((1/3.7)*B1-(5.02/A1)*LOG10((1/3.7)*B1+13/A1)))
cell C1

=POWER(1/C1,2)

Zigrang, D.J., Sylvester, N.D. (1982). "Explicit approximations to the solution of Colebrook's friction factor equation." *AIChE J.*, 28(3), 514–515. http://dx.doi.org/10.1002/aic.690280323

6. Ćojbašić and Brkić approximation-Improved Romeo et al.

$$\left.\begin{aligned}
&\frac{1}{\sqrt{\lambda}} \approx -2\cdot\log_{10}\left(\frac{1}{3.7106}\cdot\frac{\varepsilon}{D}-\frac{5}{\mathrm{Re}}\cdot A_{12}\right)\\
&A_{12} \approx \log_{10}\left(\frac{1}{3.8597}\cdot\frac{\varepsilon}{D}-\frac{4.795}{\mathrm{Re}}\cdot A_{13}\right)\\
&A_{13} \approx \log_{10}\left(\left(\frac{1}{7.646}\cdot\frac{\varepsilon}{D}\right)^{0.9685}+\left(\frac{4.9755}{206.2795+\mathrm{Re}}\right)^{0.8759}\right)
\end{aligned}\right\}$$

MATLAB:

```
function Lambda=Lambda(Re,RelEpsilon)
Lambda = (-2.*log10((RelEpsilon./3.7106)-
(5./Re).*log10((RelEpsilon./3.8597)-
(4.795./Re).*log10((RelEpsilon./7.646).^0.9685+(4.9755./(206.2795+Re))
.^0.8759)))).^-2;
```

MS Excel:

=-2*LOG10(B1*(1/3.7106)-5/A1*LOG10(B1*(1/3.8597)-(4.795/A1)*LOG10(POWER(B1*(1/7.646),0.9685)+POWER(4.9755/(206.2795+A1),0.8759))))

cell C1

=POWER(1/C1,2)

Ćojbašić, Ž, and Brkić, D. (2013). “Very accurate explicit approximations for calculation of the Colebrook friction factor.” *Int. J. Mech. Sci.*, 67, 10-13.
http://dx.doi.org/10.1016/j.ijmecsci.2012.11.017

7. Ćojbašić and Brkić approximation-Improved Seghides

$$\left.\begin{aligned}
&\frac{1}{\sqrt{\lambda}} \approx A_{14} - \frac{(A_{15}-A_{14})^2}{A_{16}-2\cdot A_{15}+A_{14}}\\
&A_{14} \approx -2\cdot\log_{10}\left(\frac{1}{3.71}\cdot\frac{\varepsilon}{D}+\frac{12.585}{\mathrm{Re}}\right)\\
&A_{15} \approx -2\cdot\log_{10}\left(\frac{1}{3.71}\cdot\frac{\varepsilon}{D}+\frac{2.51\cdot A_{14}}{\mathrm{Re}}\right)\\
&A_{16} \approx -2\cdot\log_{10}\left(\frac{1}{3.71}\cdot\frac{\varepsilon}{D}+\frac{2.51\cdot A_{15}}{\mathrm{Re}}\right)
\end{aligned}\right\}$$

MATLAB:

```
function Lambda=Lambda(Re,RelEpsilon)
A14 = -2*log10(RelEpsilon./3.71+12.585./Re);
A15 = -2*log10(RelEpsilon./3.71+2.51.*A14./Re);
A16 = -2*log10(RelEpsilon./3.71+2.51.*A15./Re);
Lambda = (A14-((A15-A14).^2)/(A16-2.*A15+A14)).^-2;
```

MS Excel:

=POWER(-2*LOG10(B1*(1/3.71)+12.585/A1)-POWER(-2*LOG10(B1*(1/3.71)+2.51*(-2*LOG10(B1*(1/3.71)+12.585/A1))/A1)--2*LOG10(B1*(1/3.71)+12.585/A1),2)/((-2*LOG(B1*(1/3.71)+(2.51*(-2*LOG10(B1*(1/3.71)+(2.51*(-2*LOG10(B1*(1/3.71)+12.585/A1))/A1)))/A1)))-2*(-2*LOG10(B1*(1/3.71)+2.51*(-2*LOG10(B1*(1/3.71)+12.585/A1))/A1))+(-2*LOG10(B1*(1/3.71)+12.585/A1))),-2)

Ćojbašić, Ž, and Brkić, D. (2013). "Very accurate explicit approximations for calculation of the Colebrook friction factor." *Int. J. Mech. Sci.*, 67, 10-13.
http://dx.doi.org/10.1016/j.ijmecsci.2012.11.017
